# Automatic quantification of perivascular spaces in T2-weighted images at 7 T MRI

**DOI:** 10.1016/j.cccb.2022.100142

**Published:** 2022-04-05

**Authors:** J.M. Spijkerman, J.J.M. Zwanenburg, W.H. Bouvy, M.I. Geerlings, G.J. Biessels, J. Hendrikse, P.R. Luijten, H.J. Kuijf

**Affiliations:** a Department of Radiology, University Medical Center Utrecht, Utrecht, the Netherlands; b Brain Center Rudolf Magnus, Department of Neurology, University Medical Center Utrecht, Utrecht, the Netherlands; c Julius Center for Health Sciences and Primary Care, University Medical Center Utrecht, Utrecht, the Netherlands; d Image Sciences Institute, University Medical Center Utrecht, Heidelberglaan 100, 3584 CX, Utrecht, the Netherlands

**Keywords:** Perivascular spaces, Centrum semiovale, Quantification, 7 tesla MRI, Machine learning

## Abstract

Perivascular spaces (PVS) are believed to be involved in brain waste disposal. PVS are associated with cerebral small vessel disease. At higher field strengths more PVS can be observed, challenging manual assessment. We developed a method to automatically detect and quantify PVS.

A machine learning approach identified PVS in an automatically positioned ROI in the centrum semiovale (CSO), based on -resolution T2-weighted TSE scans. Next, 3D PVS tracking was performed in 50 subjects (mean age 62.9 years (range 27–78), 19 male), and quantitative measures were extracted. Maps of PVS density, length, and tortuosity were created. Manual PVS annotations were available to train and validate the automatic method.

Good correlation was found between the automatic and manual PVS count: ICC (absolute/consistency) is 0.64/0.75, and Dice similarity coefficient (DSC) is 0.61. The automatic method counts fewer PVS than the manual count, because it ignores the smallest PVS (length <2 mm). For 20 subjects manual PVS annotations of a second observer were available. Compared with the correlation between the automatic and manual PVS, higher inter-observer ICC was observed (0.85/0.88), but DSC was lower (0.49 in 4 persons). Longer PVS are observed posterior in the CSO compared with anterior in the CSO. Higher PVS tortuosity are observed in the center of the CSO compared with the periphery of the CSO.

Our fully automatic method can detect PVS in a 2D slab in the CSO, and extract quantitative PVS parameters by performing 3D tracking. This method enables automated quantitative analysis of PVS.

## Highlights

• Fully automated detection of perivascular spaces on 7T MRI.
• Good correlation with manual assessments of perivascular spaces.
• Quantitative measurements of PVS characteristics: density, length, and tortuosity.

## 1 Introduction

Perivascular spaces (PVS) are fluid-filled spaces around the brain-perforating blood vessels [1], and are connected to the cerebrospinal fluid (CSF) in the subarachnoidal space. PVS can be observed on MR images, where they appear with a (visually) similar signal intensity as CSF [1]. PVS are believed to be involved in the clearance of waste products from the brain [2,3]. Furthermore, the appearance of more and/or larger perivascular spaces has been linked to aging [4], [5], [6] and is a feature of cerebral small vessel disease (SVD) [6], [7], [8]. Therefore, perivascular spaces are highly relevant when investigating the healthy and diseased brain.

Currently, PVS are mostly evaluated visually, using qualitative measures such as a rating scale for PVS counts in brain regions like the basal ganglia (BG) and the centrum semiovale (CSO) [9], [10], [11]. Actual PVS count could offer a more precise method to determine PVS load, since this results in a continuous scale rather than an ordinal scale, thereby eliminating discretization and ceiling effects. However, manual annotation is highly labor intensive, especially at higher field strengths where high numbers of PVS are observed [12]. Also, inter-rater differences remain, since for example (very) small PVS can easily be confused with motion or noise. It is illustrative that in a recent 7 T MRI study on average more than 70 PVS could be counted in a single slice from one hemisphere, making their manual assessment very labor intensive. This limits further evaluation of PVS in high resolution scans, regarding e.g. PVS asymmetry [13]. Still, even if manual PVS annotation would be feasible, it still lacks additional quantitative information. An automatic method would be easier to apply and eliminates inter-rater variability, and could be used to acquire quantitative PVS measures, such as PVS length and tortuosity.

In this work a method was developed to automatically detect and quantify PVS in the centrum semiovale. High-resolution 3D T_2_-weighted TSE scans of 50 subjects (27–78 years) were available. Automatic PVS detection was performed in an automatically determined region-of-interest (ROI) in the CSO. The method was trained and validated with manual PVS annotations that were available in a single slice in the CSO, in the right hemisphere [12]. 3D tracking of all automatically detected PVS resulted in PVS length and PVS tortuosity measurements in these subjects.

## 2 Methods

### 2.1 Data

Scans of 50 subjects (mean age 62.9 years (range 27–78), 19 male) from two earlier studies with identical 7 T MRI protocols were used. Scans of 30 participants of the PREDICT-MR study [14], and scans of 20 participants of the second Utrecht Diabetic Encephalopathy Study (UDES2) were available [15]. PREDICT-MR and UDES2 were approved by the medical ethics committee of the University Medical Center Utrecht (UMCU), and all subjects gave written informed consent. The guidelines of the Declaration of Helsinki of 1975 were followed. The PREDICT subjects were randomly recruited in waiting rooms of general practices and had no cognitive impairment. The UDES2 subjects were recruited through their general practitioners, and had no cognitive impairment. Of the 20 UDES2 subjects included in this study, 8 subjects had diabetes mellitus. The used data is described in detail by Bouvy et al. [12]. Briefly, for all subjects a 3D T_2_-weighted TSE scan acquired at 7 T was available, with 0.7 mm isotropic resolution, reconstructed to 0.35 mm isotropic. TR/TE were 3158/301 ms. Also, for all subjects a T_1_-weighted TFE scan acquired at 7 T was available, with 1 mm acquired isotropic resolution, reconstructed to 0.66 × 0.66 × 0.5 mm^3^. TR/TE were 4.8/2.2 ms. For all subjects PVS markers from an expert observer were available in the centrum semiovale, in the right hemisphere [12] of a predefined slice. This slice was located in the CSO, 1 cm above the most cranial slice where the lateral ventricles were visible. For subjects of the UDES2 study also PVS markers of a second observer were available.

### 2.2 Semiautomatic PVS detection and tracking

**Fig. 1.**
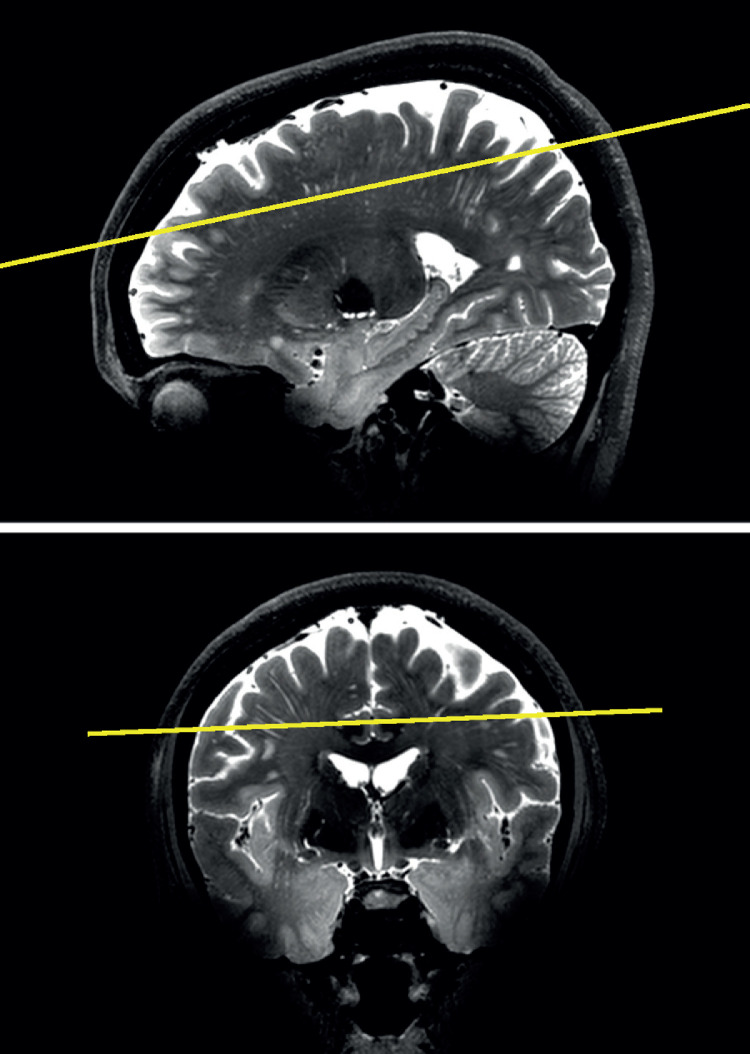
Automatically determined CSO plane (yellow) in a subject (T_2_-weighted TSE scan), in sagittal (TOP) and coronal (BOTTOM) view. The CSO plane is located 1 cm above the ventricles, and is oriented parallel to the line connecting the bottom of the genu and splenium of the corpus callosum, and perpendicular to the midsagittal plane.

The T_1_-weighted scans were used for segmentation of white matter (WM) using SPM12 [16]. The WM mask was registered to the T_2_-weighted scans using rigid registration with elastix [17]. To enhance all vessel-like structures, the T_2_-weighted scans were filtered with a vesselness filter. We used a multi-scale 3D vesselness implementation as proposed by Sato et al. [18]. Specifically, we computed 10 scales with uniform distributed sigma ranging from 0.30 to 1.00 mm (inclusive) as implemented in MeVisLab [18], [19], [20]. Subsequently, a plane was automatically positioned in the CSO, parallel to the line connecting the genu and splenium of the corpus callosum, perpendicular to the midsagittal plane, and 10 mm above the lateral ventricles. The genu and splenium, and the midsagittal plane were automatically detected as described elsewhere [21], [22], [23]. The CSO-plane was extended to a slab of 7 slices, and the CSO region-of interest (CSO-ROI) was defined as the WM in this slab. Fig. 1 shows an example of the automatically positioned CSO-plane for a subject.

For the automatic PVS detection, PVS probability maps were created based on a binary kNN (k nearest neighbor^1^) classifier [24] trained using the T_1_ and T_2_ signal intensities, and the vesselness values. A kNN classifier first builds a multidimensional feature space trained on labelled data that contains examples of the ‘foreground (i.e. PVS) and the ‘background’ (i.e. white matter). A leave-one-out approach was used: for each subject, the classifier was trained on the data of all other subjects. The training data included all manually annotated PVS points dilated by one voxel in all directions (resulting in a 3 × 3 × 3 window), and a random sample of 10% of all white matter voxels within the slab as background class. When provided with new data, the kNN classifier projects the new data into the trained feature space and looks up the k nearest neighbor example data points. K was set at 51 and the nearest neighbors were weighted by the inverse of their distance. All other parameters were kept at default settings. The final label of the new data is the distance-weighted average of the example data: either PVS or white matter, in this application. The labeling process is repeated for all pixels in the CSO-ROI to obtain their label. Next, local non-maximum suppression was applied to reduce multiple neighboring PVS pixels into a single seedpoint. All locations having a PVS probability above 0.50 were used in a bidirectional 3D tubular tracking algorithm (radius range: 0.15–0.70 mm; search depth: 3 steps, 2 angles, pruning threshold of 5; and a termination threshold of 10) [25]. PVS tracking was performed within the WM in the entire CSO. The length and other parameters of each PVS track were determined using the skeleton of each individually tracked PVS. The resulting PVS tracks shorter than 2 mm were considered false positives and tracks longer than 50 mm were considered to be physiologically unfeasible, and were therefore excluded.

For each subject the PVS count was determined. Furthermore, for each tracked PVS the PVS length and tortuosity τ (τ=L/C, with L the PVS length, and C the shortest distance between the begin- and endpoints of the PVS) were determined.

### 2.3 Validation metrics

To validate the automatic PVS detection method, the Intraclass Correlation Coefficient (ICC) and the Dice Similarity Coefficient (DSC) metrics were used. The data used in this work was acquired with 7 T MRI, which results in a much larger number of visible PVS compared with data acquired with e.g. 3 T MRI. This introduces a bias in the ICC, with higher ICC for a larger PVS count, as the ICC is a data-dependent metric [26]. The DSC takes also the PVS location into account, additionally to the PVS count, and is therefore regarded as a more reliable metric. However, the DSC value is generally (much) lower than the ICC value for rating highly-frequent noisy objects [26].

To compare the performance of the automatic method with manual PVS counting, PVS detection was performed in the exact same slice as manually selected by Bouvy et al. [12], in the right hemisphere. The automatically tracked PVS and the available manual PVS annotations in this slice were compared based on count and location, using the ICC and the DSC. This was compared with the available inter-observer ICC and DSC for the UDES2 data, between the first and second observer [12]. Furthermore the ICC between the automatically detected PVS in the slice used by the first observer and the automatically positioned plane was determined, as a measure of the dependency of the PVS count on the chosen scan section.

### 2.4 Additional quantitative PVS features

The subject images were registered with the MNI-152 brain template [27,28]. The registration method is described by Biesbroek et al. [29], and is also effective for scans of older subjects. The resulting transformation was applied to the 3D tracked PVS. The PVS density, length, and tortuosity distributions were plotted on top of the MNI-152 brain template. PVS density is computed as the average number of 3D tracked PVS points (resulting from the tubular tracking algorithm) within a 2 mm radius around each voxel in the MNI-152 brain template. Each individual transformed PVS skeleton in MNI-152 space was dilated by 2 mm, and its length and tortuosity were averaged with all other PVS tracks of all subjects in that area.

## 3 Results

Fig. 1 shows the automatically positioned CSO plane for one subject, relative to the T_2_-weighted TSE scan. The CSO-plane is located 1 cm above the ventricles, parallel to the line connecting the bottom of the genu and splenium of the corpus callosum and perpendicular to the midsagittal plane.

**Fig. 2.**
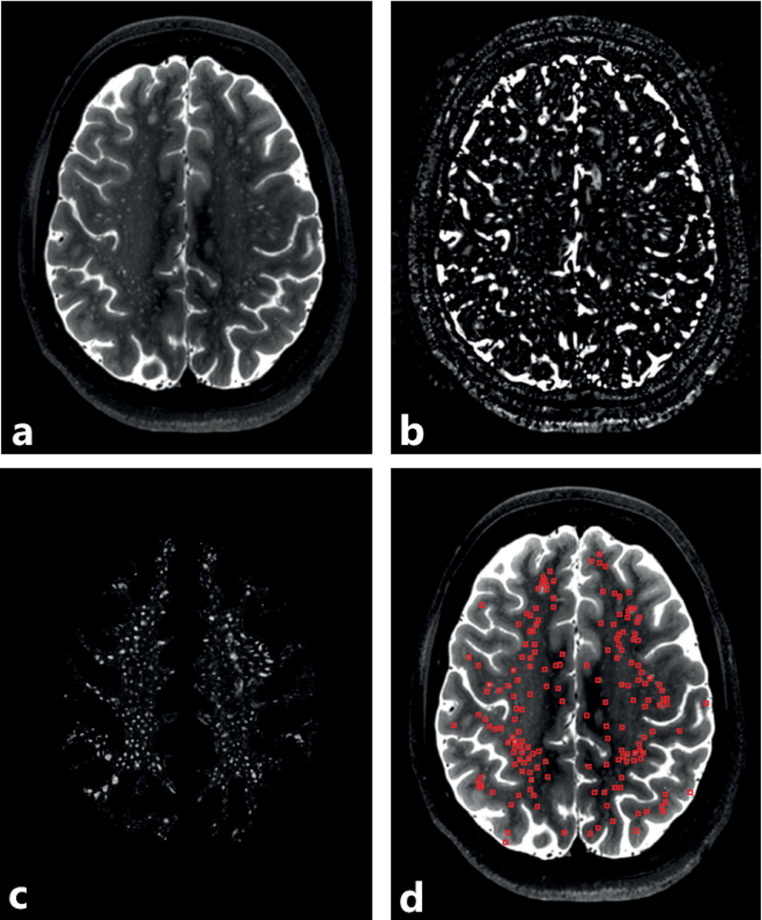
The automatically determined CSO plane from the 3D T_2_-weighted TSE volume (A), the vesselness filtered scan (B), the PVS probability map (C), and the detected PVS relative to the T_2_-weighted TSE image (D). The PVS detection was performed by thresholding the probability map (threshold = 50%).

Fig. 2 AB shows the T_2_-weighted TSE image of one subject in the middle slice of the CSO-slab, and the same image after vesselness filtering. Fig. 2 CD shows the PVS probability map resulting from the kNN classifier, and the detected PVS relative to the original T_2_-weighted TSE image.

**Fig. 3.**
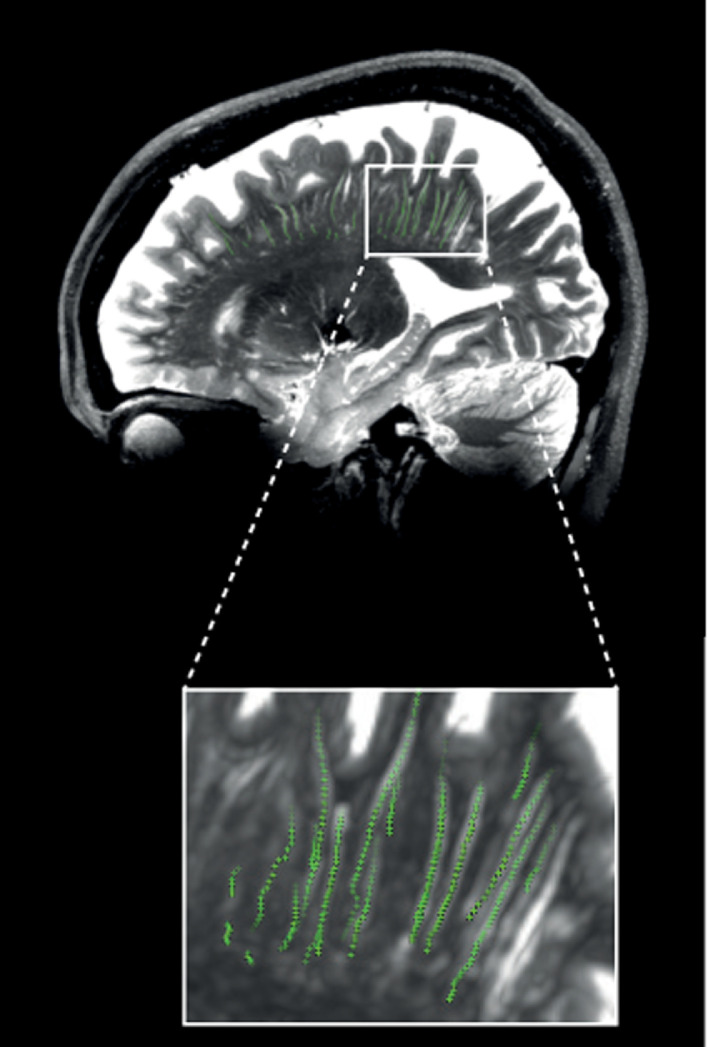
Sagittal view of the tracked PVS (green markers), relative to a MIP of a sagittal slab (thickness 4 slices) of the 3D T_2_-weighted TSE images of a subject.

For the same subject the 3D tracked PVS are shown relative to a maximum intensity projection (MIP) of a sagittal slab of the T_2_-weighted TSE scan (Fig. 3).

Total computation time was approximately three hours per subject on a standard workstation.

### 3.1 Validation of automatic PVS detection

**Fig. 4.**
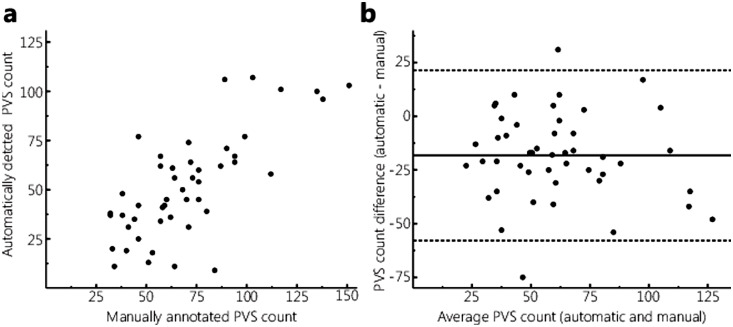
Scatterplot (A) and Bland-Altman plot (B) of the PVS count detected by the automatic method and annotated by the human observer, in the slice that was used by the human observer, in the right hemisphere. Overall, the automatic method detected less PVS than the human observer.

To assess the performance of the automatic PVS detection method, the correlation between the number of automatically detected and manually annotated PVS was determined for all 50 subjects. This was done in the exact same slice that was used by the human observer, in the right hemisphere (the left hemisphere was not taken into account by Bouvy et al. [12]). Fig. 4 shows a scatter plot and a Bland-Altman plot of the PVS count detected by the automatic method and the human observer. Overall, a smaller PVS count was found by the automatic method compared with the human observer. This can be partially contributed to the fact that the automatic method ignores the smallest PVS (length < 2 mm). The Intraclass Correlation Coefficient (ICC) (absolute/consistency) was 0.64/0.75, and the Dice Similarity Coefficient (DSC) was 0.61 between the automatically determined PVS and the manually annotated PVS. In the scans of two subjects artifacts were observed. Despite the artifacts, the method performed relatively well, although the correlation with the visual observers was below average for these two subjects.

To compare the performance of the automatic method with the performance of manual PVS annotation, correlation of the manually annotated PVS with a second human observer was determined. For 20 subjects manually annotated PVS of the second observer were available [12]. Inter-observer ICC (absolute/consistency) between the original observer and the second observer was 0.85/0.88. However, in 16/20 subjects DSC could not be determined, as different slices were selected by both observers: on average, the distance between the slices was 1.33 ± 0.98 mm (range: 0.34–4.17 mm). In four subjects both observers actually annotated the exact same slice. In these four subjects, the DSC was 0.49 (range 0.32–0.73) for the manual PVS annotations between the first and second observer. In these same four subjects, higher DSC was found for the automatic method, compared with both human observers: between the automatically detected PVS and the manually annotated PVS by the first/second observer for these four subjects, DSC was 0.64/0.62 (range 0.59–0.75 / 0.55–0.73).

Finally, as a measure of the dependency of the PVS count on the chosen scan section, the number of automatically detected PVS was compared between the slice that was used by the human observer and the automatically positioned plane. This resulted in ICC (absolute/consistency) of 0.74/0.74.

### 3.2 Additional quantitative PVS features

**Fig. 5.**
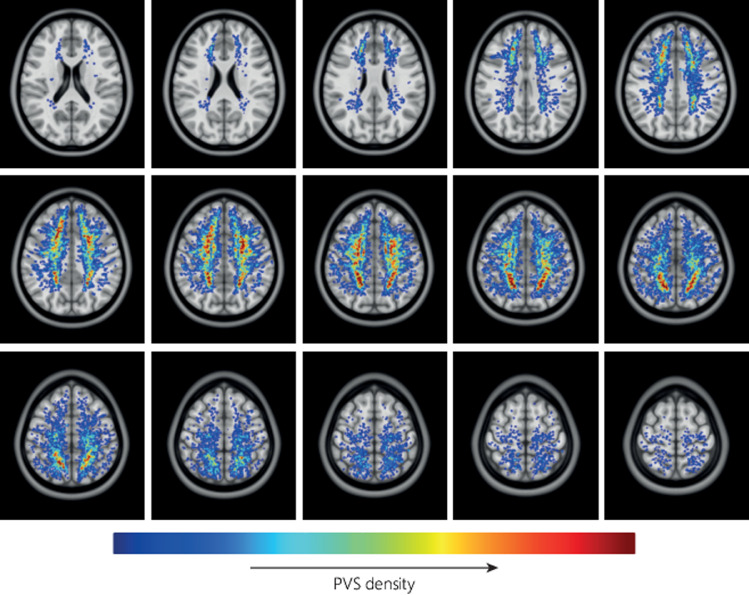
Average distribution of PVS density of all subjects, plotted in MNI space. Very similar PVS densities can be observed in both hemispheres, with two foci with high PVS density anterior and posterior in the CSO.

**Fig. 6.**
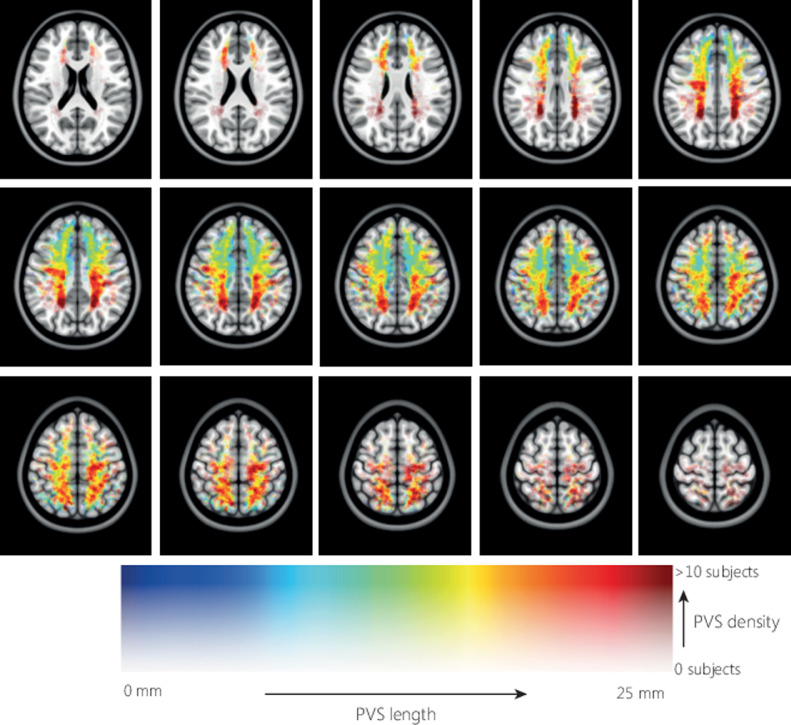
Distribution of PVS length of all subjects, plotted in MNI space. The markers were dilated to a diameter of 2 mm. The marker color indicates the PVS length, marker transparency indicates the number of PVS markers. Largest PVS lengths can be observed posterior in the CSO, shorter PVS lengths can be observed anterior in the CSO.

**Fig. 7.**
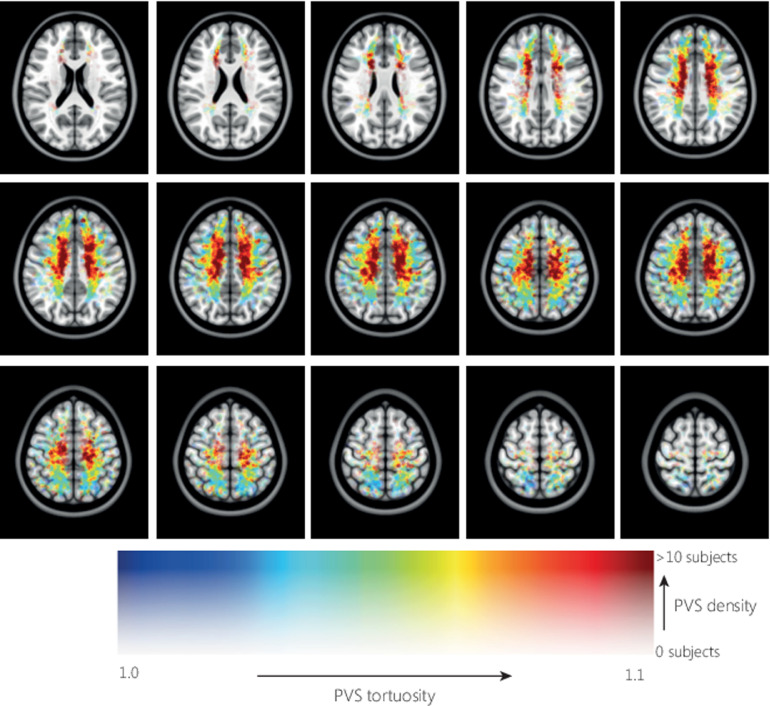
Distribution of PVS tortuosity of all subjects, plotted in MNI space. The markers were dilated to a diameter of 2 mm. The marker color indicates the PVS tortuosity, marker transparency indicates the number of PVS markers. Higher PVS tortuosity can be observed in the center of the CSO, and smallest PVS tortuosity can be observed at the periphery of the CSO and posterior in the CSO.

**Fig. 8.**
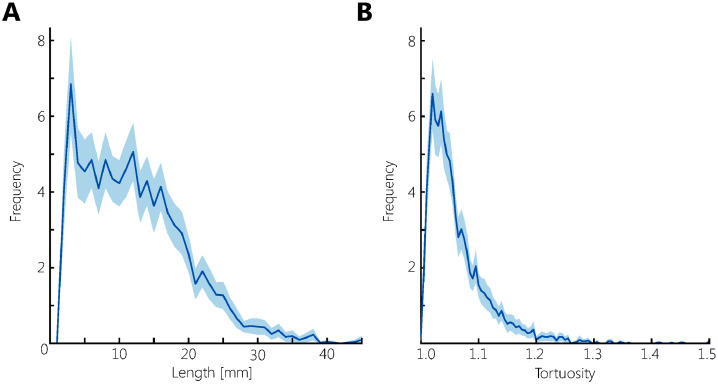
The PVS length (A) and tortuosity distribution (B) for the tracked PVS of all subjects. The line represents the average distribution, the transparent band represents the 95% confidence interval. Median PVS length is 12.1 mm (range: 2–48 mm), the median of PVS tortuosity is 1.05 (range: 1.00–1.54).

Fig. 5 shows the average distribution of PVS density relative to the MNI-152 atlas. In each hemisphere two foci of high PVS density can be observed, anterior and posterior in the CSO-slab. Fig. 6 shows the average distribution of PVS length in MNI space, the color indicates the PVS length, and transparency indicates the number of subjects. Longer PVS can be observed posterior in the CSO, and shorter PVS can be observed anterior in the CSO. Anterior in the CSO, higher PVS length are plotted in the most caudal slices compared with more cranial slices: only the longest PVS that were detected in the CSO-ROI, are located in the most caudal slices, whereas more cranially also the shorter PVS are found. Fig. 7 shows the average distribution of PVS tortuosity relative to the MNI atlas, the color indicates the PVS tortuosity, and transparency indicates the number of subjects. Higher PVS tortuosity can be observed in the center of the CSO, and smallest PVS tortuosity can be observed at the periphery of the CSO. Fig. 8 shows the average and 95% confidence interval (95% CI) distributions of PVS length and tortuosity, for the tracked PVS of all subjects.

## 4 Discussion

In this work a method was developed to automatically detect and quantify PVS in the centrum semiovale. 3D T_2_-weighted TSE images acquired at 7 T were used as input for the method. The method yields PVS measurements in an automatically detected ROI in the CSO, including count, length, and tortuosity.

A key strength of this fully-automatic method is that it is deterministic, meaning that rerunning the method yields exactly the same results. This increases repeatability and reproducibility compared with manual PVS assessment. Also, the vesselness filter that was used in this method is independent of possible signal inhomogeneities [18,19]. Therefore, possible variations in signal intensities (and thus SNR) between regions only have a limited effect on the performance of the method.

Furthermore, additionally to the PVS count, quantitative parameters (length and tortuosity) could be acquired for each PVS by performing 3D tracking of the detected PVS. Also, the large dataset enabled mapping of the quantitative parameters (PVS density, length, and tortuosity) to MNI space.

Finally, a large dataset of high-resolution scans acquired at 7 T of 50 subjects with manually annotated PVS was available for this work. This dataset enabled good validation of the automatic PVS detection method.

Our results support the hypothesis that automatic PVS detection is less sensitive to noise and/or motion compared with manual PVS annotation. Our automatic method detects the larger, well-visible PVS and ignores the smallest PVS close to the noise-level, by taking the signal intensity into account along with the vesselness value, and by applying a length restriction on the detected and tracked PVS (PVS must be at least 2 mm in length). These conditions decreased the sensitivity for noise and/or motion in the scan, and therefore resulted in a lower automatically detected PVS count compared with the manual annotated PVS. Furthermore, the manual annotations, that were available from a previous study [12], included as many PVS as possible, including the smallest PVS. As a result, the automatic method systematically detected a smaller number of PVS compared with the original manual segmentation (Fig. 4). This is also likely to contribute to the lower absolute versus consistency ICC between the automatically detected PVS count and the manually annotated PVS. On the other hand, in the four subjects where inter-observer DSC could be determined, DSC was higher for the automatic method compared with the human observer.

The performance of the automatic PVS detection method is on par with what can be expected for this specific task, i.e. counting highly-frequent and inherently noisy objects on 7 T images. Only a fraction of all PVS is sufficiently large to be distinguishable above the noise level [6,12]. This yields an inherently ill-defined difference between detectable and non-detectable PVS. Visual (and automatic) PVS rating is still possible, but a consistent method to annotate the PVS is necessary. Individual human observers have different intrinsic cut-offs for annotating an object as PVS or ignoring it as noise. Training of observers can decrease the difference between these intrinsic cut-offs, but inter-observer differences remain. Paradoxically, these inter-observer differences are amplified in 7 T scans, because of the significantly higher visibility of PVS on 7 T MRI compared to 1.5 T or 3 T. If fewer and larger PVS are visible (e.g. on 3 T MR images), the likelihood that both observers will identify the same objects is higher. On 7 T MR images, much more and smaller PVS (and PVS-like objects) are visible, which decreases the inter-observer agreement. This is not related to the performance of the observers (human or machine), but can be fully attributed to the increased difficulty of rating 7 T MR images. This was demonstrated earlier for rating cerebral microbleeds by Kuijf et al. [26]: in this work it was shown that the difference between the ICC and DSC increases for increasing object counts.

Our human observers had an inter-observer ICC of 0.85/0.88 for the PVS count, which can be qualified as “good”. However, when considering the overlap between the annotations, the DSC is only 0.49. Thus, the human observers agreed on the PVS count, but did not necessarily identify the same PVS. This demonstrates the difficulty in rating highly-frequent noisy objects on 7 T MR images: it is hard to consistently identify the same object in an image. The DSC between the automatic method and the human observers was larger than the inter-observer DSC. This illustrates that the agreement between the method and either observer is larger than the inter-observer agreement.

Several automatic methods for (semi-)automatic PVS segmentation have been proposed by different groups currently working on automatic PVS detection [30], [31], [32], [33], [34], [35], [36]. For the automatic PVS detection and tracking method that was proposed in this work a low-resolution T_1_-weighted scan (to determine the WM mask) and a high-resolution T_2_-weighted TSE scan (for PVS segmentation) were used as input, with a total scan time of approximately 12 min. This scan time is relatively short compared with other automatic PVS detection methods. For example, Park et al. [31] used two high-resolution (T_1_- and T_2_-weighted) scans acquired at 7 T, with a total scan time of approximately 22 min. Our automatic PVS detection method resulted in similar or higher correlation with human observers, compared with other automatic detection methods. Boespflug et al. [33] used a PVS detection method based on signal intensities in T_1_-weighted, fluid-attenuated inversion recovery, T_2_-weighted, and proton density data (acquired at 3 T), and filtered the detected PVS based on multiple morphological features (width, volume, and linearity). In contrast, in our method included a morphological feature (vesselness) in the detection step, thereby eliminating the need for extensive filtering after PVS detection. Boespflug et al. tested their method in a dataset of 14 subjects (age ranging between 70 and 101 years), and achieved correlation R of 0.54–0.69, relative to three raters. In our study R was 0.76 compared with the manually annotated PVS. Park et al. used a learning-approach method for PVS detection, and Lian et al. [32] used a fully convolutional neural network approach. Both methods used extensive image filtering: Park et al. used randomized 3D Haar features, and Lian et al. incorporated enhancement of the used MRI images using a non-local Haar-transform-based method, which was introduced by Hou et al. [37]. In contrast, in our work a more simple method based on vesselness value and signal intensity was used. Both Park et al. and Lian et al. compared their automatic methods with a ground truth that was determined semi-automatically, in subsets of 11 and 14 subjects, respectively. Both methods resulted in relatively high correlation relative to the ground truth, with DSC slightly above 0.7. This is higher than the DSC of 0.61 found in our work, which was derived from a dataset of 50 subjects. However, in our work the automatic method was compared with manually annotated PVS, including very small PVS, which were excluded from the ground truths used by Park et al. and Lian et al.. Also, Park et al. used young, healthy volunteers (age ranging between 25 and 37 years) and detected on average 298 PVS in the whole brain, whereas in this study also elderly subjects were included with higher PVS density (on average 106 PVS were detected in only one slice). Furthermore, the scans used in our work occasionally showed motion, which potentially decreased the overall correlation with the manually counted PVS.

In work by Hou et al. [37] 7 T MRI scans were enhanced using a method based on the Haar transform, which increased the distinguishability of PVS. Hou et al. showed that PVS detection by thresholding the scans after vesselness filtering was significantly improved by using the enhanced and denoised scans. In our work a combination of the vesselness filter with signal intensities in the T_2_-weighted scan was used for PVS detection. Incorporating Hou's scan enhancement in our automatic method may offer possibilities to further improve both detection and tracking of PVS. However, in our method only larger PVS were included (PVS shorter than 2 mm are excluded), and therefore the scan enhancement by Hou et al. is expected to offer only minor improvements for our PVS detection method.

Future methods could consider the use of (convolutional) neural networks, to replace the kNN method for detection of the PVS. For example the nnDetection framework [38] has shown promising results in detecting small objects [39]. A downside is that neural network methods usually require larger example training data sets. An alternative approach is to use convolutional neural networks to completely segment PVS at once, which was one of the tasks in the “Where is VALDO” challenge [40].

The method presented in this work was developed for PVS detection in a 2D slab, similar to commonly used visual rating scales [9], [10], [11], which enabled thorough validation using the manually annotated PVS. This is contrary to several other published (semi-)automatic methods [30], [31], [32], [33], [34], [35] where PVS segmentation is performed in the entire brain or within certain brain regions (centrum semiovale and basal ganglia). The comparison between the PVS count in the automatically determined CSO-ROI in this work and the slice used by the first observer in Bouvy et al., resulted in ICC of 0.74. This is in line with literature, where lower correlation was found between observers when different slices were used [35], and relatively low correlation was observed between PVS count in a single slice and in the whole brain [30]. Automatic slice selection, as was performed in our method, can reduce PVS count variation induced by the selected slice. However, whole-brain PVS segmentation or PVS segmentation for 3D brain regions may offer the most reliable PVS count. Therefore we aim to extend our method to a full 3D assessment in future work.

The spatial differences in PVS density, length, and tortuosity observed in this work may be related to the relatively high age of subjects in this study. Contrary to our results, Park et al. [31], found similar PVS lengths in the parietal-occipital lobe and the frontal lobe, and slightly shorter PVS in the temporal lobe, in younger subjects (25 – 37 years). Also, Park et al. found shorter PVS, up to 18 mm, compared with the PVS lengths acquired in our work. This may suggest that, in aging subjects, PVS posterior in the CSO increase in length before PVS in other regions of the CSO. It would be very interesting to further investigate such regional differences in PVS properties in relation to age and between different diseases. Furthermore, Feldman et al. have recently found asymmetry in PVS count in epilepsy patients [13]; possibly also asymmetry in PVS length and/or tortuosity can be found in diseases such as epilepsy.

In contrast to Park et al. [31], Boespflug et al. [33], Ramirez et al. [30], and Cai et al. [34], PVS diameter and volume were not determined in this work, which could be regarded as a limitation. However, the measured PVS diameter can be expected to depend strongly on the acquired resolution. Moreover, reliable diameter estimates require more than a single voxel within the PVS, limiting measurements to only the largest diameters > 1 mm [41]. Because of the small PVS diameter (normally less than 2 mm in healthy subjects) relative to the acquired isotropic resolution of 0.7 mm, estimates of PVS diameter and volume will certainly suffer from considerable errors and partial volume effects.

The main limitation of this work is that the method is currently not able to perform 3D detection. However, using a 2D slab enabled good validation with manual PVS assessment. In future work our automatic PVS detection method can be extended to a full-brain PVS detection method.

Also, manual PVS markers were used to train the method. These markers included the very small PVS, which may not be reproducible, and is expected to increase the risk of selecting false positives. To minimize the number of false positive detected PVS, a minimum PVS length of 2 mm after tracking was used, but false positive detected PVS cannot be prevented entirely.

Furthermore, occasionally a (long) in-plane perivascular space was detected more than once. Tracking of these detected points can result in multiple short PVS, rather than one long PVS track, thereby resulting in underestimated (average) PVS length. In the slices used for PVS detection in this work (almost) all PVS were almost perpendicular to the plane. Therefore the effect of in-plane PVS was very limited. Merging of the identified PVS pixels may resolve this issue for extending our method to whole-brain PVS detection.

The method was developed and evaluated on a single 7 T system, so future work is needed to evaluate its performance on other systems and on other field strengths. Most components in the method have already demonstrated robust performance on other scanners (e.g. vesselness and tubular tracking), but especially the kNN classifier might need to be retrained to operate with other data sets. The high spatial resolution of a 7 T system provides advantages for detecting and tracking PVS, but performance on lower field strength is therefore unknown. Initiatives like the “Where is VALDO” challenge [40] will likely provide valuable insights in PVS detection performance on more common field strength systems.

Finally, despite the high resolution of the scans used in this work, many small PVS cannot be detected due to their small size. Therefore, quantitative PVS measurements could only be performed for larger PVS. As PVS diameters range down to the micrometer scale [42], this will remain a challenge for all PVS detection methods that are based on anatomical images.

## 5 Conclusions

In conclusion, in this work we present a fully automatic method to detect PVS in a 2D slab in the CSO, and to extract quantitative PVS parameters by performing 3D tracking of the detected PVS. Our method shows good correlation with manual PVS assessment and has the potential to quantitatively study PVS characteristics such as density, length and tortuosity in aging and disease.

## Footnotes

1 Source: https://scikit-learn.org/stable/modules/generated/sklearn.neighbors.KNeighborsClassifier.html
